# Trustworthiness criteria for meta‐analyses of randomized controlled studies: OBGYN Journal guidelines

**DOI:** 10.1111/aogs.14942

**Published:** 2024-09-22

**Authors:** The OBGYN Editors' Integrity Group (OGEIG), Jason Abbott, Ganesh Acharya, Amir Aviram, Kurt Barnhart, Vincenzo Berghella, Catherine S. Bradley, Nancy Chescheir, Thomas D’Hooghe, Michael Geary, Janesh Gupta, Karl Oliver Kagan, Anthony Odibo, Aris Papageorghiou, Luis Sanchez‐Ramos, Donna Santillan, Elizabeth Stringer, Togas Tulandi, Susan C. Modesitt, Vincenzo Berghella

**Affiliations:** ^1^ Department of Obstetrics and Gynecology, Division of Maternal‐Fetal Medicine Thomas Jefferson University Hospital Philadelphia Pennsylvania USA

## 1 INTRODUCTION

Editors of a number of OBGYN journals have formed an OBGYN Editors' Integrity Group (OGEIG), with the aim of collaborating to improve trustworthiness in published papers.^1^ These Editors have already jointly agreed and published quality criteria for randomized controlled trials (RCTs), incorporating the guidance in their instructions to authors.^2^ The requirements for RCTs include:

- prospective registration in an international clinical trials registry platform, with registration number provided;
- statement of ethical approval, with name of approving committee;
- statement of informed consent;
- statement of adherence to CONSORT guidelines (and, ideally, provision of a CONSORT checklist to be published as supplementary material);
- and a data‐sharing statement.^2^

Meta‐analysis is a quantitative statistical technique used to combine and analyze data from the results of multiple previous independent studies on a particular topic, to derive overall conclusions or effect estimates. In general (but not exclusively), meta‐analyses are based on RCTs. The results are often used to develop standard practice or clinical guidelines. However, RCTs may be inaccurate or fabricated, leading to journal withdrawal or retraction. This article aims to expand upon the list of RCT quality criteria for authors of meta‐analyses of RCTs, so that low‐quality and fabricated studies are excluded from meta‐analyses.

## 2 MATERIAL AND METHODS

The editors in the group were invited to participate in monthly or bimonthly calls regarding trustworthiness in OBGYN publishing, with the aim of preventing publication of untrustworthy science in women's health. Using data from the published literature, including our prior work,^1^, ^2^ Cochrane guidance,^3^ the TRACT Checklist,^4^ the author instructions of the various journals, and other publications related to trustworthiness of meta‐analyses of RCTs,^5^ criteria for meta‐analyses were reviewed, reaching consensus by majority.

## 3 RESULTS

By consensus, 21 quality criteria were agreed upon by the editors. The aim is for authors to check and confirm the quality criteria for *each* identified RCT when carrying out a meta‐analysis of RCTs (Tables 1 and 2). These criteria help to identify trustworthy RCTs, and are assigned to two groups: absolute criteria and “other quality” criteria. “Absolute” trustworthiness criteria are those that, if not met, would suggest noninclusion in the main results of meta‐analyses of RCTs (Table 1). “Other quality” criteria are those that, if not met, would suggest lower quality of RCTs (Table 2). In addition, the meta‐analysis should be prospectively registered in the PROSPERO database (or a similar international, publicly accessible database, eg INPLASY; Research Registry – Registry of Systematic Review/Meta‐Analysis).

**TABLE 1:** “Absolute” RCT trustworthiness criteria.

Domain	Item	Statement	Checked?
Governance	Retraction	The RCT has not been retracted (*Check PubMed* ^7^ *and RetractionWatch* ^8^)	
	Registration	The RCT was preregistered in a publicly available international clinical trials registry prior to randomization *(for RCTs commencing ≥ 2010)*	
	Ethics	The RCT was approved by an ethics committee or an institutional review board (IRB)	
	Consort	Statement that CONSORT guidance^9^ was followed *(for RCTs commencing ≥ 2010)* In addition, it is preferred but not mandatory that the RCT includes: CONSORT algorithm/diagram CONSORT checklist (25 items)	
Outcome	Outcome variation	The primary outcome of the RCT is consistent with the stated primary outcome in the RCT registration	

*Note*: In general, if an article does not meet all criteria, this would suggest **noninclusion in the main results** of a meta‐analysis of RCTs.

**TABLE 2:** “Other quality” RCT criteria.^a^

Domain	Item	Statement	Checked?
Governance	Retraction	The article does not have an expression of concern (EoC) attached (*Check PubMed* ^7^ *and RetractionWatch* ^8^)	
	Registration	RCT registration details are included in the manuscript: Registration # Registration date Registry name and URL Date of first patient randomized (verify that date of RCT registration is before the first patient enrollment in the study)	
	Ethics	The ethics approval details appear in the manuscript: Ethics committee/IRB approval reference # Ethics committee/IRB approval date Ethics committee/IRB name Statement that all participants in the RCT gave appropriate informed consent before enrollment	
	Sample size	The published sample size is close (≥85%) to the intended sample size stated in the registry entry (*Check protocol in registry against sample size stated in manuscript*)	
		Estimated sample size is appropriately calculated (eg *power and type 1 error stated; target effect size and assumed rate in control group stated; standard deviation; any other adjustments such as the dropout or loss to follow‐up rates, justified). Post hoc analysis shows adequate sample size achieved. For pilot RCTs, sample size calculation is not needed*.)	
Author group	Number	The RCT has at least three authors	
Methodology	Selection bias	The allocation concealment is plausible and well explained (eg *two interventions but only one placebo,* i.e., *not matching for both interventions*)	
	Plausibility	The methodology is reasonable for the type of intervention (eg *blinding of participants when this is not possible, as in surgeries or invasive procedures*)	
	Randomization	Randomization method is clearly explained (eg *computer‐generated, block randomization, opaque envelopes,* etc.)	
Time frame	Recruitment	The number of actual recruited participants is reasonable in relation to eligible participants within the timeframe (eg *ideally RCT mentions total # deliveries in study centers during the study period, # eligible patients; # screened patients*)	
	Submission	The manuscript was submitted at least 3 months after the last patient completed follow‐up (*Based on manuscript submission date, last randomization date, and follow‐up date*)	
Dropout	Withdrawals and lost‐to‐follow‐up	The authors address lost‐to‐follow‐up participants, and there are some patients lost to follow‐up (*Check in CONSORT algorithm; lost‐to‐follow‐up does not apply when the study is assessing an immediate outcome,* eg *a surgical outcome*)	
	Rounded numbers	The dropout rate varies between groups, and the numbers do not appear to be artificially rounded (eg groups of 50 or 100; mostly odd or even numbers, rounded to 10, etc.)	
Baseline characteristics	Number	At least five baseline characteristics are mentioned	
	Homogeneity	Baseline characteristics do not appear perfectly balanced/do not seem implausible (eg *similar standard deviations for completely different characteristics with different means and distributions*)	
	Omission	Important prognostic factors are mentioned as baseline characteristics	
Outcomes	Effect size	The effect size is close to or similar to prior evidence (eg *if this is the only RCT showing positive results on this issue, the answer here should be “no”*)	
	Conflicting information	The information provided is in agreement with the outcomes (eg *more ongoing pregnancies than clinical pregnancies in ART studies*)	
Author group	Concerns	The authors do not have a history of previous manuscripts with retractions or expressions of concern (excluding papers withdrawn by the authors for valid—not trustworthiness‐related—reasons) If previous retractions or EoCs exist, consideration should be given to examining these authors' RCTs more closely (*Check each author on PubMed* ^7^ *and RetractionWatch* ^8^)	

*Note*: In general, if all criteria are not met, this may suggest **lower quality** of RCTs.

^a^ A risk of bias assessment with tools (e.g., Cochrane) may include assessment of allocation concealment, randomization process, etc. These do not have to be entered twice if already entered in Risk of bias assessment tool.

The consensus decision was that the abstract and primary analysis of meta‐analyses should report only trustworthy, “high‐quality” RCTs that meet all of the “absolute” criteria (Table 1). Authors of meta‐analyses are encouraged to contact RCT authors for additional information regarding the criteria in Tables 1 and 2 if the details cannot be found in the published manuscript or registered protocol. At a minimum, all coauthors of meta‐analyses should confirm at the point of submission that each included article meets the criteria included in Table 1. Individual journals may also ask authors to confirm that each article meets the criteria in Table 2, or may go further and ask authors to complete and submit a checklist for the criteria in Tables 1 and 2 for each article included in the meta‐analysis. In general, RCTs that are published as an abstract only seldom report all criteria in Tables 1 and 2, and so would often not be included in meta‐analyses. Authors could consider a secondary analysis excluding RCTs that, while meeting all “absolute criteria,” do not meet some of the “other quality” criteria. Authors of meta‐analyses should also provide all items noted in the Submission Checklist for Meta‐analyses of RCTs (Table 3). A risk of bias assessment with tools (eg Cochrane) may include assessment of allocation concealment, randomization process, etc. These do not have to be entered twice (eg in Tables 1 and 2) if already entered in Risk of bias assessment tool.

**TABLE 3:** Submission checklist for meta‐analyses of RCTs.

Checklist items	Completed/Reported ✓
All items in Tables 1 and 2 have been assessed and fulfilled	
PROSPERO details	
PRISMA Checklist	
Risk of bias assessment (e.g., Cochrane tools, etc.)	

## 4 DISCUSSION

Confirming trustworthiness of RCTs is crucial to the integrity of meta‐analyses, and assessment must begin with a thorough examination of the published RCTs being considered for inclusion. RCTs are more rigorous than other types of investigations, precisely because they are controlled. A meta‐analysis of RCTs combines those studies and extracts data to produce a pooled estimate, and tests for statistical significance. It is more robust than narrative reviews, and synthesizes the evidence rather than simply providing a review of the literature. Meta‐analysis of RCTs should meet the Cochrane criteria adopted by PRISMA (Preferred Reporting Items for Systematic Reviews and Meta‐Analyses), and include the necessary risk of bias assessment.^6^ Subgroup or sensitivity analysis should be considered when there is a high variability or heterogeneity among studies.

A meta‐analysis is useful because it may overturn or amplify results from smaller RCTs. Preregistration is an important aspect in reducing bias, ensuring that the study protocols are registered prospectively—that is, before the first participant is enrolled; changes to this plan during the course of the study can introduce bias. The International Committee of Medical Journal Editors (ICMJE) recommends mandatory prospective registration. Preregistration contributes to improving reproducibility of research, prevents duplication of efforts, and reduces the potential for bias. The most commonly used registry is ClinicalTrials.gov. Others are listed in the WHO Registry Network. The registry requires data on 24 mandatory elements of a study when preregistering. The study should follow CONSORT guidelines and the authors should have agreed to share the data if requested.

Retracted RCTs or low‐quality studies have often been included in meta‐analyses, including those with fabricated or plagiarized data. Evaluating RCTs against the governance criteria outlined in Table 1 will help to address this issue. Including risk of bias assessment of the included studies is important to evaluate the quality of the studies. Trustworthy meta‐analyses clearly describe the methods, search strategy, inclusion and exclusion criteria, data extraction procedures, and statistical analysis. If trustworthiness issues are raised, the RCT data should be shared upon request where available.

Including low‐quality articles (Table 2) in a meta‐analysis reduces the trustworthiness of the results. Trustworthy meta‐analyses mitigate publication bias through techniques such as funnel plot asymmetry, statistical tests, and sensitivity analyses. Factors that contribute to trustworthiness include transparency, allowing evaluation of the thoroughness of the meta‐analysis.

Using the criteria suggested in this paper (Tables 1, 2, 3), authors can conduct trustworthy meta‐analyses that will promote the progression of science with integrity and reliability. The results may then be applied with confidence into standard clinical practice or clinical guidance.

## CONFLICT OF INTEREST STATEMENT

The author declares no conflicts of interest.

## APPENDIX A Participating members of the OBGYN Editors' Integrity Group (OGEIG)

### A.1

All OGEIG members who participated in meetings discussing the manuscript “Trustworthiness criteria for meta‐analyses of randomized controlled studies” are listed here, with their affiliated journal/organization in parentheses: Jason Abbott (J Minim Invasive Gynecol), Ganesh Acharya (Acta Obstet Gynecol Scand), Amir Aviram (Am J Obstet Gynecol MFM), Kurt Barnhart (Fertil Steril), Vincenzo Berghella (*Am J Obstet Gynecol MFM*), Catherine S. Bradley (Am J Obstet Gynecol), Nancy Chescheir (COPE), Thomas D'Hooghe (Gynecol Obstet Invest), Michael Geary (Int J Gynecol Obstet), Janesh Gupta (Eur J Obstet Gynecol), Karl Oliver Kagan (Arch Gynecol Obstet), Anthony Odibo (Ultrasound Obstet Gynecol), Aris Papageorghiou (BJOG), Luis Sanchez‐Ramos (American Journal of Obstetrics & Gynecology), Donna Santillan (Proceedings Obstet Gynecol), Elizabeth Stringer (Int J Gynecol Obstet), Togas Tulandi (J Obstet Gynaecol Can), and Susan C. Modesitt (Gynecol Oncol Rep). [Correction added on 18 August 2025, after first online publication: the affiliation of one of the participating members of the OBGYN Editors’ Integrity Group (OGEIG) has been rectified.]
