# Correction to “Trustworthiness criteria for meta‐analyses of randomized controlled studies: OBGYN Journal guidelines”

**DOI:** 10.1111/aogs.70047

**Published:** 2025-09-03

Trustworthiness criteria for meta‐analyses of randomized controlled studies: OBGYN Journal guidelines. *Acta Obstet Gynaecol Scand*. 2024; 103: 2118–2121. https://doi.org/10.1111/aogs.14942

In the list of participating members of the OBGYN Editors' Integrity Group (OGEIG), Dr. Luis Sanchez‐Ramos' affiliation was incorrectly stated as *Am J Obstet Gynecol MFM*; it should have been given as *Am J Obstet Gynecol*.

The online version of the article has now been rectified.

We apologize for this error.

